# Functionalized Hemodialysis Polysulfone Membranes with Improved Hemocompatibility

**DOI:** 10.3390/polym14061130

**Published:** 2022-03-11

**Authors:** Elena Ruxandra Radu, Stefan Ioan Voicu

**Affiliations:** 1 Advanced Polymer Materials Group, University Politehnica of Bucharest, 1-7 Gh. Polizu Street, 011061 Bucharest, Romania; radu.elena.ruxandra@gmail.com; 2 Department of Analytical Chemistry and Environmental Engineering, Faculty of Applied Chemistry and Materials Science, University Politehnica of Bucharest, 1-7 Gh. Polizu Street, 011061 Bucharest, Romania

**Keywords:** hemodialysis, covalent functionalization, composite membranes, polysulfone

## Abstract

The field of membrane materials is one of the most dynamic due to the continuously changing requirements regarding the selectivity and the upgradation of the materials developed with the constantly changing needs. Two membrane processes are essential at present, not for development, but for everyday life—desalination and hemodialysis. Hemodialysis has preserved life and increased life expectancy over the past 60–70 years for tens of millions of people with chronic kidney dysfunction. In addition to the challenges related to the efficiency and separative properties of the membranes, the biggest challenge remained and still remains the assurance of hemocompatibility—not affecting the blood during its recirculation outside the body for 4 h once every two days. This review presents the latest research carried out in the field of functionalization of polysulfone membranes (the most used polymer in the preparation of membranes for hemodialysis) with the purpose of increasing the hemocompatibility and efficiency of the separation process itself with a decreasing impact on the body.

## 1. Introduction

**Figure 1:**
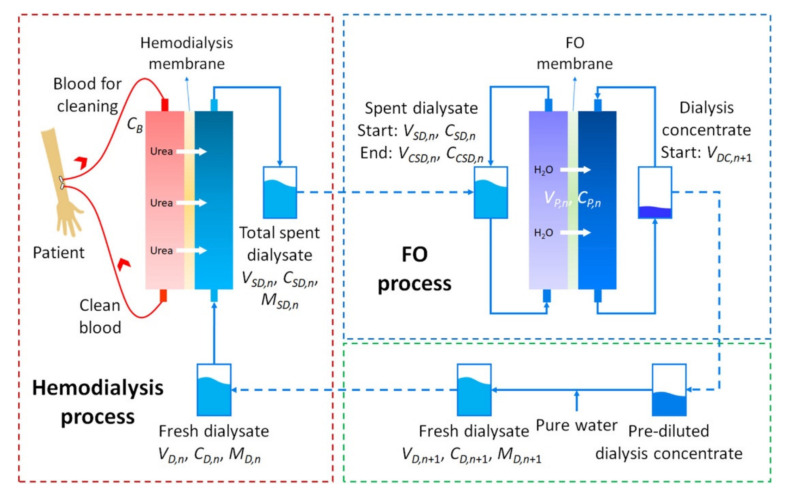
Scheme of the combination of the hemodialysis and spent dialysate recovery by osmotic dilution. A partial amount of water in the spent dialysate spontaneously moves toward the dialysis concentrate, as a consequence of the osmotic pressure gradient across the FO membrane. After FO, a certain amount of pure water is added to further dilute the dialysis concentrate. The dialysate without recovered water from the spent dialysate is used in the first hemodialysis session (reproduced with permission after [2]).

Since the beginning of their existence, humans have been preoccupied with providing for their primary needs such as food and shelter. As these necessities were resolved, increasing the quality of life and solving health problems became priorities. The advances made in scientific knowledge at the beginning of the last century led to an exponential increase in the knowledge we have and implicitly provided access to solve an increasing number of problems. One of the major health problems that found its solution at the beginning of the last century is chronic kidney dysfunction. The kidneys are those paired organs responsible for cleaning the blood by forming urine and eliminating waste from the body through it, especially the excess water, salts and metabolites resulting from the processing of proteins—urea, uric acid and creatinine. The decrease in the activity of these organs is initially indicated by the onset of acute kidney dysfunction (which can be controlled medicinally) and affects the sufferer’s life regimen, in the advanced cases causing chronic kidney dysfunction. In this case, the function of the kidneys is substituted with the help of polymer membranes for hemodialysis, which for four hours once every two days, filter the blood, removing from it what should have been removed by the kidneys in 48 h. It is estimated that currently in the world, between 4.9 and 9.7 million people need dialysis [1]. The main causes that lead to the appearance of chronic kidney dysfunction are diabetes, high blood pressure and pollution. Due to this last reason, it is assumed that the number of patients who will need this therapeutic procedure in the future will increase exponentially in the near future. The field of membranes for hemodialysis is one of the most dynamic fields, in which it is necessary to adapt both the materials used and the procedures for obtaining them. Besides the direct need for membrane materials for the therapeutic process, hemodialysis is a process that generates large quantities of water that in turn must be purified. The dialysis used during the procedure can lead in the case of a single dialysis center to the production of approximately 80 tons per year of water with a high content of urea, uric acid and creatinine, which in turn must be purified by concentrating these substances through other membrane processes, the most used at present being forward osmosis [2,3]. Figure 1 shows the separation scheme used in the hemodialysis process, as well as the scheme of further purification of the waters resulting from the therapeutic process for obtaining pure water that can be reused, as a rule, also for dialysis.

Membrane materials, initially widely used for water filtration and drinkable water production [4,5,6], have found widespread applicability in the last 50 years in other areas, such as filtering in the food industry, the energy industry [7,8], catalysis [9], sensors [10], etc. One of the areas in which the importance of membrane materials has grown exponentially lately, is biomedical sciences [11]. Thus, membranes emerged for filtering and concentrating proteins, both by manipulating porosity [12] and by the modification of the active surface, as well as by the synthesis of composite membranes. Membranes for osseointegration have already found practical use especially in stomatology where they are used to favor the integration of metal implants into the bone [13,14,15], while other composite membrane materials are used in tissue engineering to obtain various scaffold-type structures [16,17,18,19].

Hemodialysis membranes represent the most important class of membranes for biomedical applications and the second largest field for the applicability of membranes produced in the world at present, the first being desalination [20]. The quality conditions that these materials must meet are related both to the efficiency of the separation and to the biocompatibility character of the synthesized final material. Unlike other materials used in bioengineering, the hemocompatibility requirement is a mandatory character for these materials.

**Figure 2:**
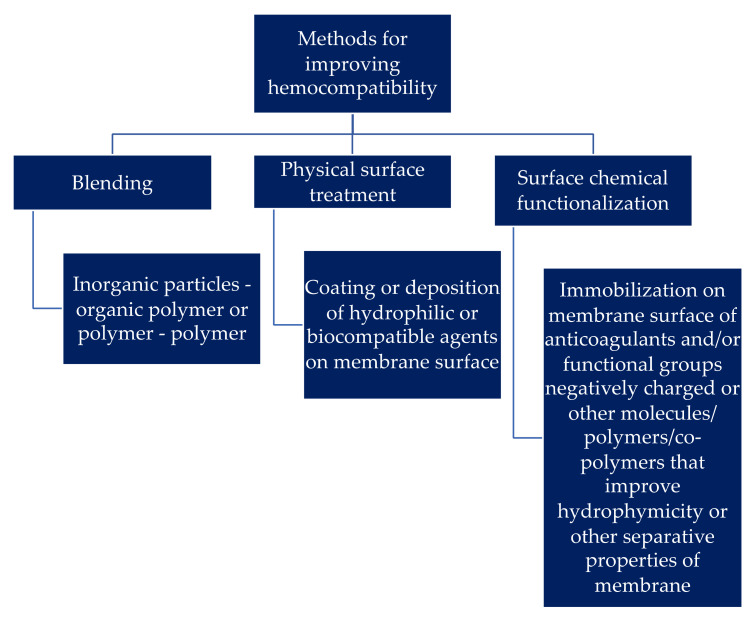
Schematic representation of most common methods for improving hemocompatibility.

Many review papers have been published in the field of hemodialysis over the years due to the high practical interest in this field. The subjects presented are divided into many areas under the same domain. The medical facilities and the management of dialyzed patients are very important concerns, since the procedure requires logistics and consumables for every patient once in two days [21]. This is one of the reasons due to which, in terms of management and logistics there is a large increase in scientific community’s interest for developing machines and technical solutions for home care [22,23,24,25]. This remains for the moment an impossibility despite the efforts for solving this. Cardiovascular implications of this medical procedure, such as medical conditions and complications associated with kidney failure [26,27] or vascular access for procedure itself [28], were subjects for a large number of studies published as review papers [29]. Environmental contamination as a main source of kidney failure has been also extensively investigated and presented in review papers [30,31]. From the perspective of materials science, the main interest is credited to polymeric membranes used for the procedure. In comparison with other medical applications for polymeric membranes (such as drug delivery systems, for examples), these membranes must have high efficiency for the separation of certain species (urea, uric acid and creatinine), but the interaction with others must be reduced as much as possible (e.g., other proteins or elements from blood or active pharmaceutical compounds used as complementary treatment for kidney failure or for the treatment of other organs affected by this) [32]. The preferentially used polymer for this application is polysulfone, used for more than five decades, due to its remarkable chemical and physical properties [33]. Recent advances in the field of new polymer synthesis investigated the possibility for at least partially replacing polysulfone, without success until now, but generated a large amount of research results and valuable data [34,35,36]. The control of biofouling [37] represents a key point for membrane characteristics. For this reason, in relation to the engineering of the hemodialysis process, some reviews has been published that present surface phenomena and interactions and also technical solutions to address hydrophilicity and hydrophobicity, repulsion or attraction forces between ions and membrane surface and also explanations from a physical chemistry point of view for hydration layers and friction-reduction properties [38,39,40]. In terms of hemocompatibility, several methods can be used for improving this property in hemodialysis membranes [41]. A summarization of these methods is presented in Figure 2.

The most common methods are blending (inorganic particles with polymeric membrane or polymers/co-polymers with polymeric membrane, in which the filler increases the surface hydrophilicity), physical surface treatment (in order to deposit at the surface of the membrane hydrophilic or biocompatible layers under a physical process that turns the hydrophobic surface into a hydrophilic one) and surface chemical functionalization. Chemical functionalization allows the immobilization of various chemical species that improve the separation performances, increase hydrophilicity or improve hemocompatibility of the hemodialysis membranes. The novelty of the present review is given by the new approach in the field of studies related to polysulfone functionalized membranes for hemodialysis from the perspective of functionalization reactions at the surface of the membrane that improve hemocompatibility. Reactions conditions and the influence of immobilized molecules over performances of separation and hemocompatibility are presented and some future trends in the field of functionalized polysulfone membranes for hemodialysis are also discussed at the end of the review.

## 2. Polysulfone Functionalized Membranes for Hemodialysis

Hemocompatibility and anticoagulant properties are the most significant qualities that need to be considered when developing advanced hemodialysis membranes [42]. Biomaterials that come into contact with blood should not initiate the process of blood clotting by adsorption of blood proteins on the surface of the biomaterial, leading to the formation of the thrombus. It was found that the hydrophobic surface character of these biomaterials could lead to the adherence of plasma proteins to the hydrophobic surfaces forming plasma clotting [43]. In the last decade, many materials were studied for their hemocompatibility, such as polyvinyl alcohol (PVA) [43,44], cellulose triacetate (CTA) [43,45], polymethylmethacrylate (PMMA) [38,46], polyacrylonitrile (PAN) [47,48], polysulfone (PSF) [9,20,49], polyethersulfone (PES) [43,50,51] and polyamide (PA) [46,52]. Polysulfone (PSF) is a thermoplastic polymer used in the biomedical field as a dialysis membrane due to physicochemical properties, such as thermal stability, chemical resistance, decent mechanical strength, great processability and biocompatibility [43].

The drawback of PSF is having a hydrophobic nature, which could be conducive to protein adsorption, platelet adhesion and clotting enzymes, which directly leads to thrombosis [38]. The functionalization of PSF surfaces changes the hydrophilicity of the surfaces, leading to an increase in the hemocompatibility and anticoagulant properties of the PSF membrane [41,53]. The functionalization of the PSF could be achieved at every step of the forming phase of the polymers, such as the functionalization of PSF in solution followed by membrane synthesis or formation, and functionalization of the surface of PSF membrane [54]. Further, in functionalization, functional groups could be used, such as small compounds that could be linked directly to the PSF or by using different linker molecules and macromolecules by the grafting of chemical functions onto an aromatic ring followed by immobilization using a linker molecule [49,55]. The PSF does not have any vacant functional groups and in order to perform functionalization, additional functional groups must be added by grafting onto the aromatic rings, such as the introduction of a sulfonate group into the polymer structure through electrophilic substitution via sulfonation [47]. The addition of the sulfonic group increases the membrane’s permeability, hydrophilicity, hemocompatibility, anti-fouling behavior and antimicrobial properties [56,57]. The sulfonation treatment is capable of influencing the morphology of PSF membranes, such that neat PSF membranes have a smoother surface and sulfonated PSF presents a significant increase in the roughness of the surfaces [34]. The sulfonated group will have a negative charge [1].

Aydemir Sezer et al. [58] presented the sulfonation treatment of PSF that could replace the heparin-based functionalization of PSF via the introduction of the sulfonic acid group, SO_3_H, into the structure of a molecule using a chlorosulfonic agent as a sulfonating agent. A core—shell electrospun structure based on PSF core and sulfonated PSF shell was investigated. The sulfonation treatment of PSF leads to an increase of the hydrophilicity of PSF indicating the improvement of blood compatibility due to hydrophilic groups on the surface of sulfonated PSF. The mechanical properties, hemocompatibility and biocompatibility properties of sulfonated PSF are improved in comparison with those of neat PSF [58]. In this study, Mahlicli et Altinkaya [59] modified PSF with alpha-lipoid acid (ALA), which is an antioxidant obtained from the reduced form of dihydrolipoic acid (DHLA), in order to obtain a hemodialysis membrane. Before preparing the support membrane, the PSF was modified via sulfonation in order to induce negatively charged groups (SO_3_^−^). ALA was immobilized onto the hemodialysis membranes used in order to prevent hemodialysis-induced oxidative stress. The described immobilization method was based on site-specific binding of a carboxylic group of ALA to an ammonium group of the anchoring polyelectrolyte layer—PEI—through electrostatic interactions. It was reported that the best antioxidant effect was seen in the case of ALA placed between two layers of PEI. In addition, reduced stress oxidation, protein adsorption and the platelet activation of the membranes were also observed [59]. Chien et al. [60] described polysulfone modification through sulfonation with sulfuric acid, decreasing the contact angle from 91.3° to 87.2° and the deposition of polyelectrolytes. Moreover, the obtained composites of PSF and the deposition of poly(acrylic acid) (PAA)/poly(allylamine hydrochloride) (PAH) multilayer films were studied for hemocompatibility through the determination of the number and morphology of adhered platelets. The results showed that the addition of the PAA and PAH layers increased the extension of platelet spreading [41]. Aydemir Sezer et al. [58] presented the sulfonation of PSF with chlorosulfonic acid resulting in a decrease of the contact angle in comparison with pristine PSF, from 133° ± 13° for pristine PSF to 125° ± 12° for sulfonated PSF. Xie et al. [61] reported the influence of sulfonated PSF with different degrees of sulfonation (20%, 30% and 50%), which was used as an additive for polyvinyl chloride (PVC). The PVC/SPSF membrane composites present an improvement in the permeability and antifouling properties of the PVC. It was shown that the degree of sulfonation of PSF influences the topology and the roughness of the obtained membranes, directly proportional with the degree of sulfonation. The increased roughness is a consequence of increasing pore size due to the larger size of the polymer lean region during the liquid–liquid phase separation leading to the larger pore size with the increased sulfonation degree. The reported hydrophilicity of sulfonated PSF via air bubble contact angle measurements was increased due to the surface segregation of sulfonate groups resulting an antifouling surface for organic foulants. BSA was used in order to study protein adsorption on the membrane’s surface. This has resulted in a decreased adsorption due to the increased hydrophilicity of the composite membranes [61].

**Figure 3:**
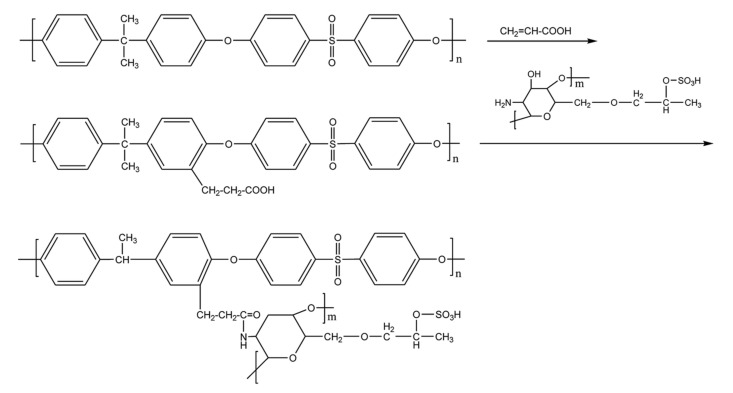
The steps of the modification of the PSF membrane (reproduced with permission after [64]).

Functionalization of PSF with sulfonated hydroxypropyl chitosan leads to an increase of the membrane’s biocompatibility and antimicrobial properties due to the presence of chitosan through a Schiff base reaction carried out between the attached acetaldehyde and ortho-aminophenol (OAP) or meta-aminophenol (MAP) of PSF [62]. Firstly, chitosan was treated with propylene/isopropanol oxide for hydroxypropyl grafting, followed by sulfonation with formamide/chlorosulfonic acid in order to obtain hydroxypropyl chitosan sulfonate. After the functionalization, the hydrophilicity was improved, resulting in better anticoagulant properties and antimicrobial properties, which recommend it as membrane for hemodialysis [63]. Tu et al. [64] modified PSF with covalently grafted acrylic acid and sulfonated hydroxypropyl chitosan for better hemocompatibility and anticoagulant properties (Figure 3). The acrylic acid was first grafted on PSF by adding the –COOH functional group, which will react with sulfonated hydroxypropyl chitosan. In comparison with the Schiff base reaction previously used in the functionalization of PSF with sulfonated hydroxypropyl chitosan, this time, covalently grafted acrylic acid and sulfonated hydroxypropyl chitosan were used. The results showed a decrease of the contact angle from 86° for neat PSF to 22° for the modified membrane with sulfonated hydroxypropyl chitosan and acrylic acid, leading to an improved hydrophilicity of the functionalized membrane, which results in an improved performance against protein contamination. The bovine serum albumin (BSA) adsorption was lower after the grafting with both acrylic acid and sulfonated chitosan due to the increased hydrophilicity. Ganj et al. [65] functionalized PSF by grafting the acrylic acid via free graft polymerization. After the functionalization, the contact angle of the composite PSF—acrylic acid membrane was reduced by 30%. Further, the flux recovery ratio was increased by 32.2%, which could indicate that the modified membrane has antifouling properties [65].

**Figure 4:**
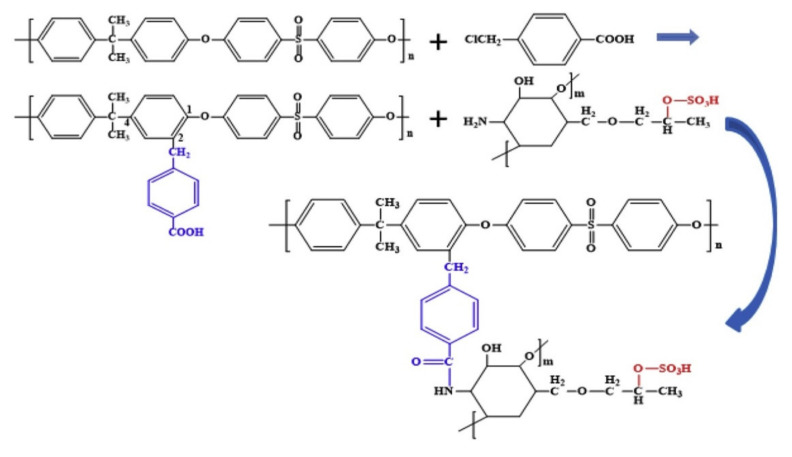
The modified polysulfone membranes obtained by grafting 4-(chloromethyl)benzoic acid and sulfonated hydroxypropyl chitosan (reproduced with permission after [66]).

Yan et al. [66] functionalized PSF with sulfonated hydroxypropyl chitosan and 4-(chloromethyl)benzoic acid in order to improve hemocompatibility. The 4-(chloromethyl)benzoic acid was grafted on PSF via the Friedel—Crafts alkylation reaction, providing available carboxyl groups, followed by the grafting of sulfonated hydroxypropyl chitosan via esterification (Figure 4). The functionalized membrane presented increased hydrophilicity and remarkable hemocompatibility, and the hemolysis rate decreased after functionalization. The contact angle decreased after grafting, from 86° for neat PSF to 59° after functionalization through the addition of carboxyl groups into sulfone after adding 4-(chloromethyl)benzoic acid. The reaction time of PSF functionalized membrane with 4-(chloromethyl) benzoic acid and sulfonated hydroxypropyl chitosan had a decreasing effect on the contact angle, such that, after 12 h of reaction time, the contact angle decreased up to 47° and, after 24 h of reaction time, the contact angle decreased up to 32°. These results showed an exceptional increase of the hydrophilicity, which could lead to an improved hemocompatibility.

**Figure 5:**
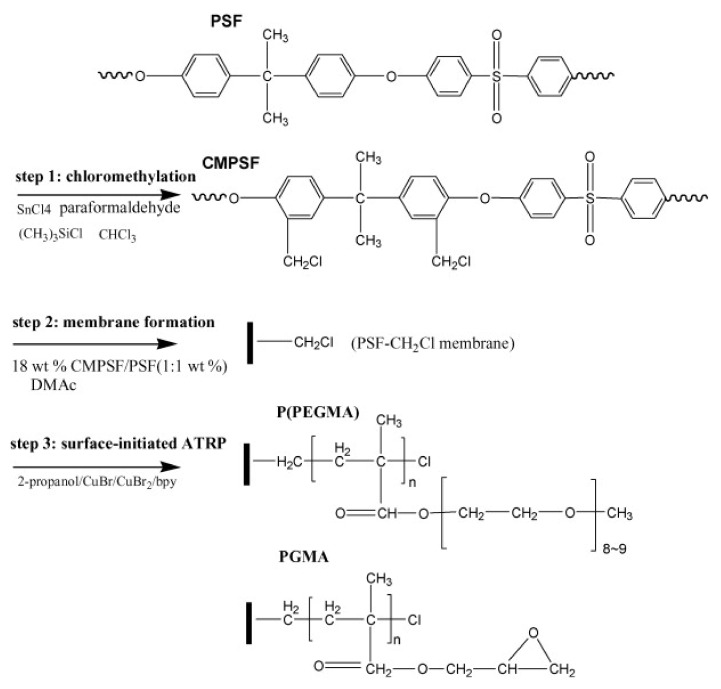
Process of surface-initiated ATRP from the polysulfone membrane (reproduced with permission after [69]).

**Figure 6:**
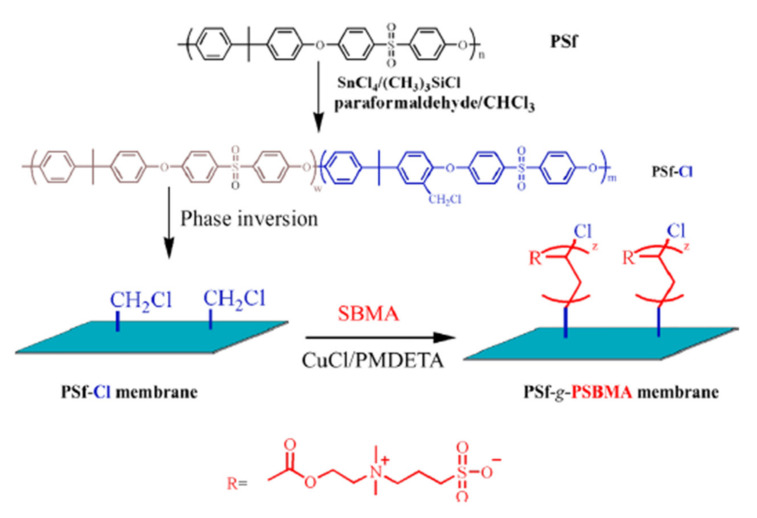
Scheme illustration of the preparation of PSF-g-PSBMA membrane (reproduced with permission after [70]).

In addition to sulfonation, another modification method of the PSF is chloromethylation, which will induce the appearance of numerous functional groups on the PSF in order to increase hydrophilicity, such as hydroxyl group (–OH), azide group (–N_3_), amino (–NH_2_), carboxyl (–COOH) and sulfo (–SO_3_H) groups [54,67]. Through chloromethylation of the PSF, a surface benzyl chloride group is introduced as an active ATRP initiator [68]. The biocompatibility of PSF is improved by the grafting of negative carboxyl and sulfonic groups. Dong et al. [69] grafted poly(poly(ethylene glycol) methyl ether methacrylate) and poly(glycidyl methacrylate) on chloromethylated polysulfone in order to increase the antifouling properties (Figure 5). After grafting, the BSA absorption decreased from ~50 to ~5 µg/cm^2^. Yue et al. [70] described PSF functionalization with zwitterionic poly (sulfobetaine methacrylate) (PSBMA) via SI-ATRP. Initially, the PSF was chloromethylated, and afterward, the sulfobetaine methacrylate (SBMA) monomer was grafted on polysulfone (PSF-g-PSBMA). The roughness of the functionalized membranes increased and contact angle values decreased after functionalization, thus the chloromethylated PSF contact angle slightly decreased up to 66°. A significant decrease of the contact angle was observed in the case of PSF-g-PSBMA, with a value up to 25°, due to sulfobetaine groups, which could form a hydration layer via electrostatic interaction in addition to the hydrogen bond (Figure 6) [70]. Liu et al. [71] present the chloromethylation of PSF in order to prepare the anion-exchange membranes (AEMs), which are polymer electrolytes that are able to conduct anions such as SO_4_^2−^, OH^−^ and Cl^−^. The AEM structure, properties and morphologies are controlled by chloromethylation conditions.

**Figure 7:**
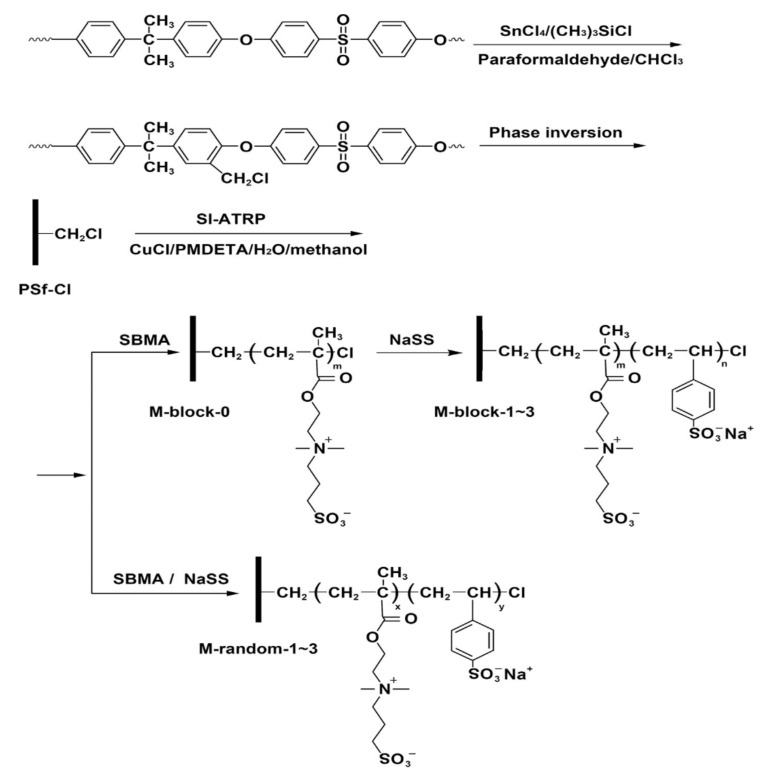
The preparation of PSF—Cl membrane, and P(SBMA-b-NaSS) and P(SBMA-co-NaSS) grafted membranes (reproduced with permission after [73]).

The zwitterionic polymers, such as poly(sulfobetaine methacrylate) (PSBMA), were grafted from a chloromethylated PSF membrane through surface-initiated atom transfer radical polymerization (SI-ATRP), in order to reduce polymer adsorption and overcome platelet adhesion, but lightly prolonged clotting [72]. The zwitterionic polymers present both positive and negative charge on the same side chain maintaining neutrality, which makes them an excellent inhibitor for the adhesion on the membrane surface of the plasma protein [72]. The chlorine groups (–Cl) were attached to the PSF via the chloromethylation reaction and used as initiators for the ATRP reaction using as reagents paraformaldehyde, chlorotrimethylsilane ((CH_3_)_3_SiCl) and tin (IV) chloride (SnCl_4_), followed by the synthetization of block zwitterionic polymer via SI-ATRP (Figure 7). The modified PSF with grafted zwitterionic showed better antifouling properties and hemocompatibility in comparison with neat PSF [73]. Maggay et al. [74] proposed a PSF membrane modified with zwitterionic polymer, a copolymer made of styrene and 4-vinylpyridine units via a dual-bath procedure, and studied the antifouling properties of the modified membrane. The results showed that the biofouling was reduced by 87% in comparison with the pristine PSF membrane after incubating the membranes with *E. coli* and with a 90% reduction of biofouling in the case of whole blood.

**Figure 8:**
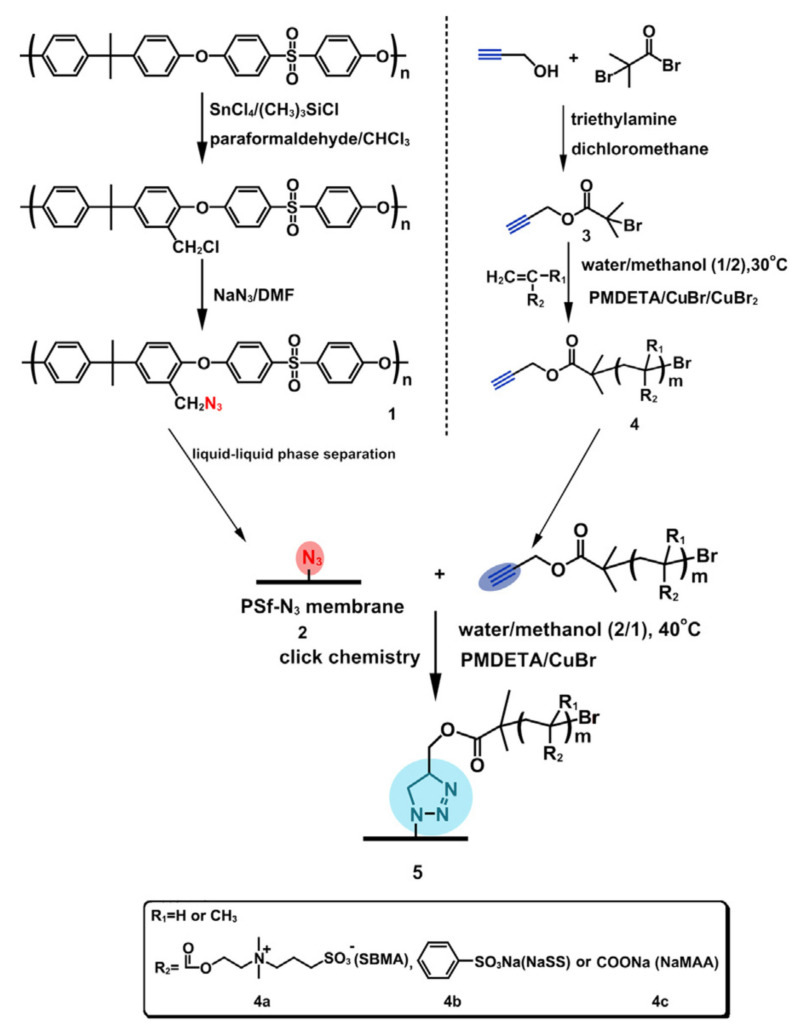
Synthesis of functionalize azido-polysulfone, alkynyl-functionalized polymers and grafted polysulfone membrane via click chemistry (reproduced with permission after [49]).

In addition, the combination of zwitterionic polymers and the negatively charge given by the sulfonic and carboxyl groups, increases the hydrophilicity of the PSF, resulting in a good antifouling property and hemocompatibility. Xiang et al. [49] also proposed zwitterionic polymers of poly(sulfobetaine methacrylate) (PSBMA) and negatively charged polymers of poly(sodium p-styrene sulfonate) (PNaSS) and/or poly(sodium methacrylate) (PNaMAA) to functionalize PSF membranes via click chemistry in one step (Figure 8). The resulting polymers were obtained via ATRP and linked at the surface of the azido-functionalized PSF membrane via click chemistry. The obtained functionalized membranes presented increased hydrophilicity, due to the decrease of contact angle values, good resistance to protein adsorption and platelet and bacterial adhesion. Further, the addition of negative charge improved the anticoagulant properties due to the synergistic effect of the sulfonic and carboxyl group.

**Figure 9:**
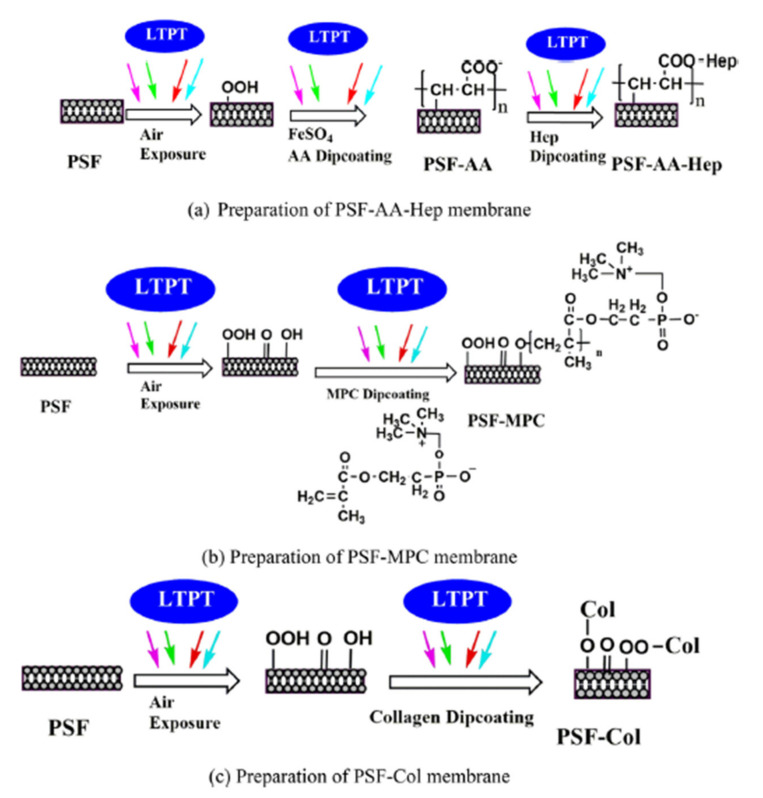
Modification process of surface grafting (reproduced with permission after [77]).

A surface modification of PSF via on plasma treatment was reported [75]. The surface hydrophilicity was improved via plasma treatment, such that a decrease of the contact angle was observed on increasing the oxygen plasma treatment time. Through plasma treatment, oxygen-containing polar groups were introduced to the membrane surfaces resulting in an increase in hydrophilicity behavior on the PSF membrane [76]. The low-temperature plasma treatment (LTPT) was extensively used in membrane surface modification. Zheng et al. [77] reported a successful surface modification of the PSF membrane via LTPT and grafting acrylic acid (AA) and PEG, 2-methacryloyloxyethyl phosphorylcholine (MPC), heparin and collagen (Figure 9). Zhang et al. [78] described a surface modification of the PSF via ammonia–oxygen (NH_3_–O_2_) plasma treatment and found that the hydrophilicity of membrane was improved in comparison with pristine PSF membrane. Furthermore, the antifouling properties of the modified PSF membrane surface via low-temperature water vapor plasma was studied. After plasma modification, oxidation occurred, leading to improving wettability of the PSF membrane [79].

Anticoagulation properties are crucial when speaking about the hemocompatibility of hemodialysis membranes. The functionalization of PSF membrane with different anticoagulant compounds was studied in order to improve hemocompatibility. Compounds such as heparin [80], polyethylene glycol (PEG) [81], polyvinyl pyrrolidone (PVP) [82] and sulfobetaine metacrylate (SBMA) [49] have been studied for PSF functionalization [66]. Moreover, the heparin-modified PSF membrane was studied as a solution for upsurging the hemocompatibility and anticoagulant properties of the PSF membrane [64,83,84]. Heparin is a linear, sulfated glycosaminoglycan produced by mast cells, having repeating monomeric disaccharides of uronic acid and glucosamine in a 1,4-linkage, and with a three-dimensional structure in a helical form [85,86]. Heparin is used as an anticoagulant agent, administrated to prevent dialyzer and circuit clotting [87,88,89,90]. Heparin is used worldwide and is approved by the Federal Drug Administration (FDA) for the treatment of deep vein thrombosis and pulmonary embolism [86,89].

**Figure 10:**
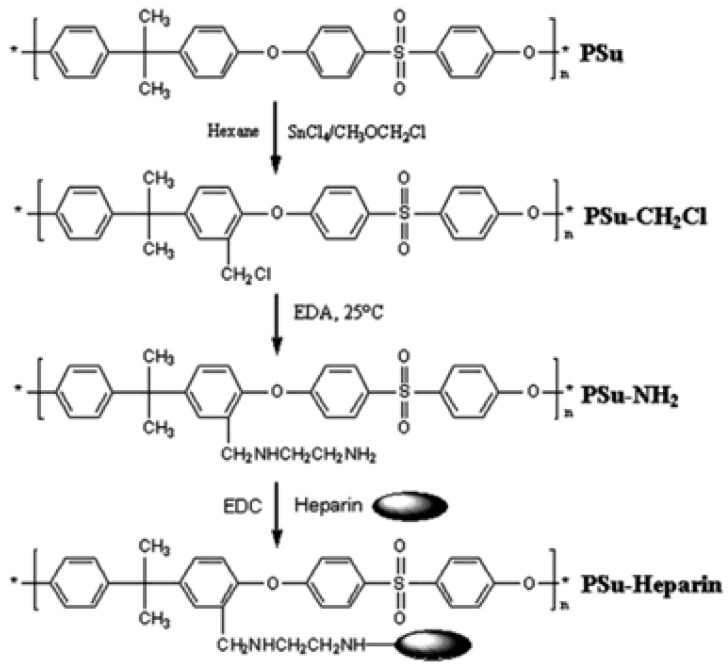
Scheme of the chemical activation of PSF film (reproduced with permission after [84]).

The heparin immobilization on PSF membrane is called a heparization treatment, and it is obtained via chemical amination, electrostatic self-assembled layers and plasma treatment [91]. Mostly, heparin immobilization is obtained through chemical amination by a chemically activated PSF membrane of the amino group, followed by heparization treatment. Li et al. [91] studied the covalent immobilization of heparin onto PSF using atmospheric pressure glow discharge (APGD) in order to chemically bind heparin molecules on the PSF surface. Huang et al. [84] described covalent immobilization of heparin on the PSF membrane for the particular adsorption of low-density lipoprotein (LDL). For PSF functionalization, the activation with consecutive chloromethyl ether and ethylenediamine treatments were needed in order to obtain available functional groups linked to heparin (Figure 10). After heparin immobilization, the hydrophilicity of the PSF membrane was improved. Furthermore, compared to the pristine PSF membrane, the heparin functionalized PSF membrane greatly enhanced the adsorption of LDL.

The drawback of the use of heparin is the fact that the only source of heparin is from animal tissues, leading to a possible risk of virus contamination and adverse effects, thrombocytopenia for long-term treatments and hemorrhages for patients with a large administrated dose of heparin [85]. It was also shown that heparin only inhibits plasma-free thrombin but does not inhibit clot-bound thrombin [47].

**Figure 11:**
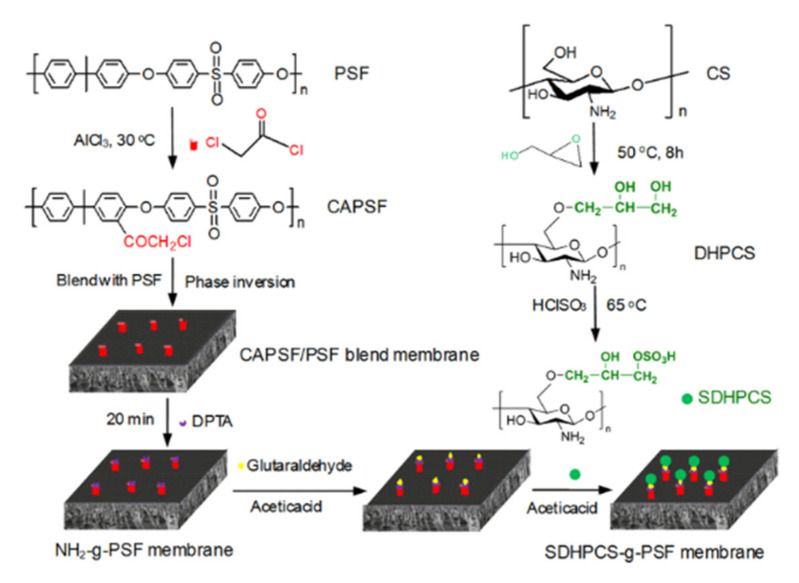
The preparation process of SDHPCS-g-PSF membrane (reproduced with permission after [93], Scheme 1).

Lately, heparin-mimic compounds with a better control over structure, sulfation and purity have been developed. Lin et al. [92] developed PSF functionalized with sulfonated citric chitosan, which has a structure and groups similar to heparin to improve the hemocompatibility of PSF. Sulfonated citric chitosan was obtained via *N*-acylation and sulfonation to take the negative carboxyl and sulfonic groups and attach them to chloroacylated PSF, which previously was treated by introducing –COCH_2_Cl groups into the PSF, followed by the preparation of chloroacylated membranes via the phase separation method. The obtained membranes present outstanding hydration capacity, which highly diminishes the amount of protein adsorption. Wang et al. [93] proposed an increase of hydrophilicity and anticoagulation properties of the PSF membrane by grafting onto the PSF surface a heparin-like polymer, sulfonated dihydroxypropyl chitosan (SDHPCS), via alkalization of chitosan, etherification and sulfonation (Figure 11). The reactivity of PSF was achieved by using chloroacetyl chloride. The results showed that the structure of functionalized PSF is not destroyed by the functionalization, but in comparison with pristine PSF, functionalized PSF has increased hydrophilicity, lower BSA adsorption and enhanced blood compatibility than before.

Liu et al. were the first to propose vorapaxar-modified polysulfone (VMPSF) for increased hemocompatibility. Vorapaxar is a protease-activated receptor 1 (PAR1) that is able to stop the cascade of reactions required for clotting. The composite membrane was obtained via the liquid–liquid phase inversion method. The functionalized membrane with vorapaxar presents a decrease of the contact angle values, leading to an increase in hydrophilicity [47]. Qi et al. [94] suggested a functionalization of PSF with resveratrol as an antioxidant compound for an improved hemodialysis-induced oxidative stress. Resveratrol is a plant-based extracted antioxidant used as an inhibitor of the oxidation reaction caused by free radicals. The functionalization is obtained via an immersion precipitation phase inversion method. The functionalized PSF membrane with resveratrol has increased hydrophilicity; improved water flux of the membrane; excellent and strong free radical scavenging ability; improved resistance of protein adhesion and excellent urea (90.33%), creatinine (89.50%) and lysozyme retention (74.60%). Another natural antioxidant used in PSF functionalization is silibilin. The functionalization was obtained via the immersion precipitation phase inversion method. The introduction of silibilin in the PSF matrix improves the hydrophilicity, antioxidant properties and hemocompatibility [95].

Nanofunctionalization is a novel direction in obtaining PSF with greater properties developed by the addition of different nanoparticles, such as TiO_2_ [96], SiO_2_ [97], Al_2_O_3_ [98], iron oxide [99] and carbon-based nanoparticles [100,101,102]. PSF functionalized with carbon nanotubes has been previously reported. Firstly, the PSF was chloromethylated, followed by functionalization with different types of nanotubes, single walled (SWNT) and double walled (DWNT). The functionalized membrane presents a large pore size and relatively small compact structure, which could be used in blood filtration as it favors the flow [100]. Mahat et al. [101] reported polysulfone functionalization with carbon quantum dots in order to improve the hydrophilicity of pristine PSF and antimicrobial properties. Zheng et al. [103] proposed an increase of antifouling properties of polysulfone via carbon nanoparticles obtained from agricultural waste of corn stalks. The modified membranes improved hydrophilicity by increasing the pore size and modified roughness leading to improved membrane permeability and antifouling properties.

Polysulfone functionalization with clay nanoparticles was studied. Recently, Ouradi et al. [104] reported a nanofunctionalization of PSF with poly(acrylonitrileco-sodium methallyl sulfonate) copolymer (AN69) (which is a hydrophilic material) and montmorillonite clay (MMT) for hemodialysis applications. It was reported that the nanocomposite membrane presents hydrophilic properties, good thermal sterilization resistance, better water permeability and a good capacity of protein adsorption. AN69 induces a negative charge on the PSF surface, and MMT reduces the free volume between PSF chains leading to increased permeability due to the rise of hydrophilic character. The addition of MMT also increases the thermal stability of the membrane, promoting a great thermal sterilization resistance [104,105,106]. Likewise, clay-polysulfone nanocomposites were developed by Ma et al. [107] via the phase inversion method. *N,N*-Dimethyl acetamide (DMAc), deionized water and PEG 400 were used as solvent, coagulant and pore-forming agent, respectively. The nanocomposite membrane displayed an asymmetric structure, with increased hydrophilicity due to the addition of MMT.

Mansur et al. [108] functionalized polysulfone membrane with silica nanoparticles (SiO_2_) in order to remove the protein uremic toxin by adsorption and alpha mangostin (α-mangostin), which is an antioxidant bioactive compound extracted from mangosteen pericarp. After functionalization, the obtained composite membranes were more hydrophilic in comparison with pristine polysulfone membrane, having an increased permeability and biocompatibility. Song et al. [109] modified polysulfone with poly(1-vinylpyrrolidone)-grafted silica nanoparticles (PVP-g-silica). The contact angle values of the composite membrane decreased with the increase of PVP-g-silica content, inducing a hydrophilic behavior. The antifouling properties were reported as being improved in comparison with those of the polysulfone/nanosilica composite membrane.

In the past decade, the functionalization with iron oxide has attracted attention due to its favorable properties for biomedical applications, such as biocompatibility, chemical stability and nontoxicity [110,111]. The usage of iron oxide improves the separation performance. Said et al. [112] reported the obtaining of polysulfone membrane functionalized with iron oxide for better biocompatibility and being able to remove the middle molecule uremic toxin effectively. It was shown that even though a high concentration of iron oxide was added, it displayed a reduction of protein adsorption and platelet adhesion while maintaining normal blood coagulation time and admissible complement activation [112]. Unfortunately, iron oxide nanoparticles have a tendency to form aggregates. For better dispersion, it was reported that the addition of citric acid could improve iron oxide dispersion in the polymer matrix [113,114,115].

TiO_2_ nanofunctionalization is used due to the super hydrophilicity behavior of TiO_2_ nanoparticles, UV resistance, antimicrobial activity and biocompatibility [116]. Nevertheless, many applications of polysulfone functionalized with TiO_2_ are in the wastewater purification field, due to its electrochemical properties, chemical stability, non-toxicity, powerful anti-oxidizing power [117,118,119].

Lately, the reduction of uremic toxin retention through functionalized PSF was studied [56,108,120,121,122]. The uremic toxins are organic or inorganic substances that are accumulated in the body fluids of patients with acute or chronic kidney disease and impaired kidney function [123]. The accumulation of these uremic toxins has a dangerous effect on the physiological function, leading to the appearance of intoxication, resulting in the deterioration of the clinical conditions [43,124]. The uremic toxins, such as urea and creatinine, are water-soluble compounds with a small molecular weight (<0.5 kDa), which are conventionally removed using dialysis [43,124,125,126]. Activated carbon, hydrated zirconium-oxide, hydrated zirconium-phosphate and activated aluminum silicate were used as the absorbent for removing uremic toxins [52,121]. Abidin et al. [56] proposed a dual-layer hollow-fiber membrane based on PSF/amino-silanized poly(methyl methacrylate) for uremic toxin separation. These sandwich-structured membranes were formed by using two layers of PSF and polyvinylpyrrolidone (PVP), which is used for pore formation, and an outer layer from silanized PMMA with 3-(aminopropyl) triethoxysilane (APTES), for the introduction of adsorption properties to the composite membrane. After silanization, the adsorption capacity was improved, with the composite membrane being able to remove urea and also to filter larger lysozyme molecules. Further, Abidin et al. [121] reported upgraded composite membranes based on PSF/activated carbon (AC) as an inner layer and PSF/PMMA as an outer layer, developed for urea and creatinine removal. The addition of AC increases the creatinine adoption.

Chen et al. [127] obtained a PSF-block-polyethylene glycol (PEG) membrane via nonsolvent-induced phase separation (NIPS). The PEG blocks are covalently bound to the PSF blocks, resulting in a composite membrane with an increased permeability (the pure water permeability (PWP) was reported being 225 L m^−2^ h^−1^ bar^−1^), compared with PSF functionalized with TiO_2_ nanoparticles and chitosan (PWP being 31 L m^−2^ h^−1^ bar^−1^) [128] and PSF functionalized with TiO_2_ nanoparticles and HEMA (PWP being 115 L m^−2^ h^−1^ bar^−1^) [129], but presenting similar BSA retention (almost 90%) compared with the aforementioned composite membrane. Said et al. [112] reported that the PSF/Fe_2_O_3_ composite membranes obtained a great BSA and urea retention (99.9% and 82%), good lysozyme retention (46,7%), but a lower a permeability (PWP being 110.47 L m^−2^ h^−1^ bar^−1^). Mansur et al. [108] reported that the addition of SiO_2_/alpha-mangostin nanoparticles increased urea and creatinine clearance (92.48% and 87.71%), compared with neat PSF membrane.

The removal of the uremic toxins with a larger molecular weight is still a challenge. Kohlová et al. [122] proposed removal of the middle-size uremic toxins (molecular weight 0.5–15 kDa) via PSF functionalized with hydrophilic additives, such as polyethylene glycol (PEG) and polyvinylpyrrolidone (PVP). The functionalized PSF membrane allowed a selective molecular separation, attributed to the dense top skin layer of the membrane, with a partial removal of middle-size molecules, and bovine serum albumin (BSA) rejection close to 100%. Table 1 summarizes the previously presented functionalized polysulfone membranes and their main properties.

## 3. Conclusions and Future Perspectives

Some of the recent approaches in the field of polysulfone functionalized membranes for hemodialysis from the perspective of functionalization reactions at the surface of the membrane that improve hemocompatibility have been presented in this review. Reactions conditions and the influence of immobilized molecules over performances of separation and hemocompatibility were presented with an accent on the surface chemistry conditions and improved properties that have been modified for blood filtration applications.

Future perspectives in relation to membranes for hemodialysis can be divided into several categories.

1. The nature of the polymer for the manufacture of membranes. At the beginning of the research to obtain dialysis membranes, the most used polymers were cellulose derivatives, especially nitrocellulose (which was found in large quantities due to its use as an explosive powder). With the discovery of polysulfone, its physical and chemical properties established it as the main polymer for obtaining membranes for hemodialysis. However, in the current context of the circular economy and the use of as many materials of natural origin as possible, we could see a return of green polymers and an intensification of the research on the use of these polymers, such as cellulose derivatives, chitosan, alginate or even starch. The current methods of synthesis of membrane materials and the possibilities of unconventional chemical modification, such as reactions in plasma or laser surface changes, can solve in this time many of the technological challenges that seemed impossible to solve in the 1950s–1960s.
2. Modification of the membrane surface to improve the hemotoxic character. The wide range of molecules that are continuously developed in the pharmaceutical industry, as well as the increasingly easy access to different methods of membrane functionalization on a scale industrial, could ensure in a reasonable time horizon, the use of more and more advanced membranes. The current problem is not the existence of technological solutions but the cost price that would be reached if these solutions were applied. Quantitatively, hemodialysis membranes are the second application for membranes produced worldwide, and an uncontrolled cost would have cascading effects from influencing capacities of production, up to the costs of the health system. Innovations in technology and chemical engineering will be able to solve such problems, allowing us to obtain functionalized membranes with other molecules, which will make synthesis easier.
3. Development of a niche field—one-day hemodialysis. In addition to treating chronic kidney failure, hemodialysis finds wider and wider applications in the purification of waste and the removal from the body of elements or substances whose concentration in the body increases following accidental poisoning. This category includes heavy metals, an organic substance used as intermediates in various syntheses or other substances from niche industries, which can reach the body following an accident. The challenges related to this type of application are far from solved. In the case of heavy metal retention, the problem is not the synthesis of a membrane that retains that heavy metal, but the fact that the membrane should retain the metal alone without removing from the blood other cations of biological interest (such as Na^+^, K^+^).
4. Coupling of hemodialysis with other biomedical processes. Patients who suffer from chronic kidney dysfunction usually have other associated conditions. A great challenge lies in obtaining membranes for hemodialysis that also have the ability to release pharmaceutically active substances, so that, during the medical procedure of blood filtration to be carried out, the treatment of associated diseases can be managed simultaneously, thus increasing the quality of life of these patients. This would be especially beneficial for those who suffer from a form of cancer for which chemotherapy is required. In addition, the release of analgesics would make the procedure more bearable, especially by alleviating the inflammatory effects that occur and are maintained for several hours after.

## Figures and Tables

**Table 1:** Functionalized membrane and induced properties.

	Membrane	Properties	Ref.
1	Sulfonated polysulfone	Hemocompatibility Biocompatibility	[58]
2	Acrylic acid and sulfonated hydroxypropyl chitosan functionalized polysulfone	Anticoagulant properties Antifouling properties Hemocompatibility Hydrophilicity	[64]
3	Sulfonated polysulfone/PVC	Permeability Antifouling properties Hydrophilicity	[61]
4	Alpha-lipoic acid (ALA) functionalized polysulfone	Antioxidant activity Antifouling properties	[59]
5	4-(chloromethyl)benzoic acid and sulfonated hydroxypropyl chitosan functionalized polysulfone	Hemocompatibility Biocompatibility Antifouling property	[66]
6	Chloromethylated polysulfone functionalized with poly(ethylene glycol)monomethacrylate (PEGMA) and 2-hydroxyethyl methacrylate (HEMA)	Antifouling properties	[68]
7	Zwitterionic copolymers of P(SBMA-b-NaSS) and P(SBMA-co-NaSS) functionalized polysulfone	Antifouling property Hemocompatibility Resistance to platelet adhesion Anticoagulant property	[73]
8	Zwitterionic polymer of poly(sulfobetaine methacrylate) (PSBMA) functionalized polysulfone	Antifouling property Hemocompatibility Cytocompatibility	[70]
9	Zwitterionic polymer of poly(sulfobetaine methacrylate) (PSBMA), negatively charged polymers of poly(sodium methacrylate) (PNaMAA) and/or poly(sodium p-styrene sulfonate) (PNaSS) functionalized polysulfone	Hydrophilicity Antifouling property Good resistance to protein adsorption, platelet adhesion and bacterial adhesion Anticoagulant property	[49]
10	Acrylic acid (AA) with heparin, 2-methacryloyloxyethyl phosphorylcholine (MPC), and collagen functionalized polysulfone	Hemocompatibility	[77]
11	Ammonia–oxygen (NH_3_–O_2_) plasma-treated polysulfone	Hydrophilicity Antifouling property	[78]
12	Plasma functionalized polysulfone	Wettability	[79]
13	Chlorodimethyl ether and ethylenediamine functionalized polysulfone	Hydrophilicity Selectivity	[84]
14	Sulfonated citric chitosan functionalized polysulfone	Hemocompatibility Anticoagulation properties	[92]
15	Heparin functionalized polysulfone	Hydrophilicity Hemocompatibility	[91]
16	AN69/MMT functionalized polysulfone	Hemocompatibility Hydrophilicity Good capacity of protein adsorption Thermal stability Permeability	[105]
17	Resveratrol functionalized polysulfone	Hydrophilicity Free radical scavenging properties Resistance to protein adhesion Hemocompatibility Antioxidant properties	[94]
18	Silibilin functionalized polysulfone	Antioxidant properties Hydrophilicity Hemocompatibility	[95]
19	Polyamide/SiO_2_ functionalized polysulfone	Excellent stability Hydrophilicity	[97]
20	Carbon quantum dot functionalized polysulfone	Hydrophilicity	[101]
21	AN69/clay composite functionalized polysulfone	Permeability Hydrophilicity Thermal stability	[105]
22	Montmorillonite functionalized polysulfone	Hydrophilicity Thermal stability Tensile properties	[106]
23	Iron oxide nanoparticle functionalized polysulfone	Hemocompatibility Biocompatibility	[112]
24	TiO_2_—graphene oxide functionalized polysulfone	Antifouling Antibacterial	[119]
25	PSF-activated carbon and PSF/PMMA	Good uremic toxin adsorption Hydrophilicity	[121]
26	Polysulfone-block-poly (ethyleneglycol)	Hydrophilicity Great permeability Antifouling property	[127]

## Data Availability

Not applicable.
